# Screening Zinc Protoporphyrin-Forming Lactic Acid Bacteria to Replace Nitrite in Meat Products

**DOI:** 10.3390/foods13233808

**Published:** 2024-11-26

**Authors:** Qianhui Yang, Zhiqiang Feng, Qian Chen, Haotian Liu, Qian Liu, Fangda Sun, Baohua Kong

**Affiliations:** College of Food Science, Northeast Agricultural University, Harbin 150030, China; yang_qianhui1@163.com (Q.Y.); ffcyfzq@163.com (Z.F.); chenqianego7@126.com (Q.C.); liuht920@neau.edu.cn (H.L.); liuqian@neau.edu.cn (Q.L.); sunfangda@neau.edu.cn (F.S.)

**Keywords:** lactic acid bacteria, zinc protoporphyrin, ferrochelatase, meat color

## Abstract

Seventeen strains of LAB (lactic acid bacteria) were screened for their ability to form ZnPP (zinc protoporphyrin) by measuring fluorescence intensity. Three strains (*Weissella viridescens* JX11, *Weissella viridescens* MDJ8, and *Lactobacillus pentosus* Q) exhibited notable ZnPP-forming ability. The ferrochelatase enzyme activity of *W. viridescens* JX11 was significantly higher than that of the other two strains (*p* < 0.05). The three selected strains were then inoculated into minced meat to observe their effect on color development and quality properties. The a*-values of the bacteria-inoculated groups were significantly higher than those of the control group and lower than those of the nitrite group (*p* < 0.05). The visible bright red color of the inoculated groups was stronger than that of the control and inferior to the nitrite group, especially in cooked minced meat. The fluorescence intensities in inoculated groups were significantly higher than those of the control and nitrite groups (*p* < 0.05). The UV–Vis absorbance data at 417 nm indicated that inoculated groups exhibited higher absorbance compared to the control group (*p* < 0.05). These results indicate that high ZnPP-forming bacteria can enhance the color of meat products and these have certain potential to replace nitrite in meat products.

## 1. Introduction

Meat color significantly influences consumers’ purchasing decisions. The coloring of meat products during processing and manufacturing considerably affects the acceptability of meat and meat products. Conventional meat products use nitrite as a curing agent due to its ability to develop a pinkish color and flavor, enhance antioxidant properties, and inhibit the growth of spoilage and pathogenic bacteria [1,2]. However, nitrate and nitrite are precursors of carcinogenic N-nitrosamines, posing health risks [3]. Hence, their addition is strictly regulated (not exceeding 0.15 g/kg), and there is a growing consumer demand for nitrate-free meat products with the increasing awareness of healthy diets. Thus, finding alternatives to nitrite is a notable challenge for the meat industry.

Meat color is related to pigment proteins such as myoglobin (Mb), hemoglobin (Hb), and trace-colored metabolites, with Mb playing a major role in color formation [4]. Acceptable Mb pigments in meat products can be classified into iron porphyrin myoglobin or zinc porphyrin myoglobin, based on the type of metal ion in the center of the heme [5]. Numerous researchers have explored nitrite substitutes, including plant-based alternatives, biological colorants, compound formulation, and organic acids [6,7,8,9,10], among which microbial fermentation substitution has garnered attention as a potential method.

Zinc protoporphyrin (ZnPP) was first found in Parma ham, a traditional Italian dry-cured ham, which is known for its stable red color without added nitrite or nitrate [11]. The mechanism of ZnPP formation has been widely studied, and three such mechanisms have been widely accepted: (1) The non-enzymatic reaction: zinc ion replaces ferrous ion in heme under anaerobic environments, hindered by the presence of oxygen, nitrite, or high temperature [12,13]; (2) enzymatic reaction: this is initiated by endogenous enzymes named ferrochelatase (FECH), whose activity decreases during the maturation of dry-cured meat products and varies in different muscle tissues and conditions [14,15]; (3) bacteria-induced enzyme reaction: Hb and hemoglobin carrier proteins serve as iron sources for bacteria, facilitated by oxidative cleavage of Hb by heme oxygenase, which results in the release of iron [16,17].

Recent studies have reported that bacteria, such as *Carnobacterium divergens*, *Serratia liquefaciens*, *Lactococcus lactis*, *Leuconostoc mesenteroides*, *Pseudomonas fluorescens*, and *Enterococcus faecium*, can form ZnPP and improve the color of meat products [18,19]. Kauser-Ul-Alam et al. [20] found that sausages inoculated with *Lactococcus lactis* subsp. cremori exhibited a bright red color. Kauser-Ul-Alam et al. [21] screened ZnPP-forming food-grade lactic acid bacteria (LAB) from various sources and evaluated their ability to enhance the color of meat products. The results revealed that 13 LAB had the ability to form ZnPP in minced meat with salt, resulting in an improved bright red color; and *Lactococcus lactis* subsp. cremoris, *Lactobacillus plantarum*, and *Leuconostoc lactis* were selected as candidates. Wu et al. [22] isolated *Leuconostoc mesenteroides* subsp. IMAU:8067 from vacuum-sealed sliced ham portions, and noted its ability to produce ZnPP. Kong, Deng, Cai et al. [23] showed that the combination of *Leuconostoc mesenteroides* subsp. IMAU:80679 and ascorbic acid led to a high level of ZnPP formation and revealed that ascorbic acid combined with *L. mesenteroides* subsp. enhances the oxidation reduction and color improvement, and could be a potential alternative to substitute nitrite in fermented sausages.

The objective of this study was to screen ZnPP-forming bacteria and explore their potential to replace nitrite in meat products. The work was performed first on the laboratory level and then on real meat product conditions. In this study, 17 species of edible LAB were screened for high ZnPP-forming bacteria in an aseptic broth model system by measuring the fluorescence intensity. The intracellular and extracellular FECH activity of the three high ZnPP-forming bacteria was determined. Subsequently, their color-enhancing ability in a minced-meat model was evaluated by analyzing the instrumental color, visual images, pH, fluorescence, and absorption spectroscopy at different fermentation times.

## 2. Materials and Methods

### 2.1. Materials and Chemicals

Table 1 summarizes information regarding the LAB obtained from various food sources. In this study, 3 kg of fresh porcine longissimus lumborum was purchased from the local market (Harbin, China) within 24 h of slaughter. Samples were stored in a chilled container with crushed ice and moved to the lab. The obvious connective tissue and fat were removed from the meat samples. Myoglobin (from equine skeletal muscle), penicillin G potassium salt, streptomycin sulfate salt, gentamicin sulfate, adenosine 5′-triphosphate (ATP), ZnPP, and protoporphyrin IX (PPIX) were purchased from the Yuanye Bio-Technology Co., Ltd. (Shanghai, China). Triton X-100 was purchased from Sigma-Aldrich (St. Louis, MO, USA). Ascorbic acid, paraffin oil, acetone, ethanol, zinc chloride (ZnCl_2_), ethylenediaminetetraacetic acid (EDTA), sodium chloride (NaCl), potassium chloride (KCl), tetrahydrofuran (THF), glycerol, tris (hydroxymethyl) aminomethane (Tris), and hydrochloric acid (HCl) were purchased from Solarbio Science & Technology Co., Ltd. (Beijing, China). All reagents and chemicals used in the experiment were of analytical grade.

### 2.2. Construction of ZnPP-Formation Model System

#### 2.2.1. Preparation of Bacterial Culture

Seventeen strains of LAB were used in this experiment, and their basic information is presented in Table 1. The most strains employed in this study partially originate from early food sample collections conducted by our laboratory, except for *Lactobacillus pentosus* Q and *Lactobacillus pentosus* S. These strains underwent meticulous processes of selective culture, purification, and rejuvenation, followed by rigorous screening and identification through multiple steps such as 16S rRNA sequencing and phenotypic characterization. All strains were double-verified based on both genetic traits and phenotypic features, exhibiting excellent fermentation properties and being carefully stored at the College of Food Science, Northeast Agricultural University, China. On the other hand, *L. pentosus* Q and *L. pentosus* S were sourced from the authoritative Shanghai Bioresources Collection Center, which boasts diverse potential applications, including in-silage preparation. Throughout the experimental procedures, we strictly adhered to the Personal Protective Equipment (PPE), sterilization, and aseptic protocols to ensure the safety and purity of the strains. Therefore, we can confidently affirm that these LAB strains are entirely safe and reliable for experimental applications.

Each strain was inoculated separately (2%, *v*/*v*) into sterile De Man Rogosa Sharpe (MRS) broth at 37 °C, and the bacterial solution was then diluted to 10^8^ CFU/mL.

#### 2.2.2. Preparation of the ZnPP-Formation Model System

The ZnPP-formation model system was constructed according to the method proposed by Wakamatsu, Okui, Ikeda, Nishimura, and Hattori [24] and Kauser-Ul-Alam et al. [21] with some modifications. To reduce microbial contamination, the surface of the fresh meat was trimmed by 0.5 cm using a sterilized knife, and the inside was minced using a sterilized meat grinder (BJRJ-82, Expro Industrial Co., Ltd., Jiaxing, China). The sample was then homogenized using a sterilized homogenizer (T25 digital ULTRA-TURRAX, IKA Equipment Co., Ltd., Staufen im Breisgau, Germany) at 6000 rpm at room temperature with distilled water to prepare a 35% pork homogenate solution containing 5% NaCl. The ZnPP-formation model system consisted of 1.8 mL of 35% pork homogenate, 0.9 mL of 0.3% myoglobin solution enriched with 0.09% ascorbic acid, and 0.3 mL of broth inoculated with a specific LAB (final concentrations: 21% pork homogenate, 3% NaCl, 0.09% myoglobin, and 2 × 10^7^ CFU/mL). An uninoculated group served as the control group. To ensure the sterile state of the control group, 0.3 mL of antibiotic solutions (penicillin G potassium (140 μg/mL), streptomycin sulfate salt (500 μg/mL), and gentamicin sulfate (100 μg/mL)) were added. The model system was overlaid with sterilized paraffin oil to shield it from atmospheric oxygen. All mixtures were incubated in the dark at 25 °C for 72 h.

#### 2.2.3. Measurement of ZnPP-Formation Ability

Fluorescence intensity was used to quantify the ZnPP formation. After three days of cultivation at 25 °C, ZnPP extraction and fluorescence intensity measurement was performed as described by Adamsen, Moller, Parolari, Gabba, and Skibsted [25] with minor modifications. Specifically, the model system solution was extracted with three volumes of ice-cold acetone for 30 min at 4 °C, followed by centrifugation at 10,000× *g* for 10 min under the same temperature conditions. The supernatant was used to measure the fluorescence intensity with a multifunctional enzyme marker (INFINITE M200 PRO, Tecan Trading Co., Ltd., Shanghai, China) in 96 microplates (*E*_x_/*E*_m_: 420/590 nm), representing the amount of ZnPP formation. Extractions were conducted in the dark to minimize ZnPP degradation.

### 2.3. Measurement of Bacterial FECH Activity

#### 2.3.1. Pretreatment of Bacterial Extracellular and Intracellular Solutions

Based on the fluorescence intensity results, three high ZnPP-forming bacteria were screened from 17 LAB species for FECH activity analysis. These strains (400 μL) were inoculated into sterile MRS broth (20 mL, 1.0 × 10^7^ CFU/mL), incubated at 37 °C for 16 h, centrifuged at 10,619× *g* for 10 min at 4 °C, and the resulting supernatant was harvested for extracellular FECH activity assessment. The precipitate was mixed with an equal volume of sterile distilled saline and sonicated using an ultrasonic homogenizer to disrupt cell walls (SCIENTZ-IID, Scientz Biotechnology Co., Ltd., Ningbo, China). The ultrasound mode involved a cycle of 2 s on and 2 s off for 15 min at 450 W, and sonication was carried out in an ice water bath. The sonicated bacterial solution was centrifuged under the same conditions and then used to measure the intracellular FECH activity. 

#### 2.3.2. Determination of FECH Activity

FECH activity was measured based on the fluorescence intensity, using the method described by Parolari, Benedini, and Toscani [26] with minor modifications. Specifically, each bacterial supernatant from MRS broth or precipitate (100 μL) was combined with 50 μL ZnCl_2_ (800 μM) in Tris-HCl buffer (360 mM, pH 8.0), 40 μL ATP (25 mM) in 20% NaCl (*w*/*v*) and 10 μL PPIX (1 mM) in the same buffer. For the blank, 70 μL EDTA (50 mM) was added to the mixture. Incubation occurred in the dark at 37 °C for 45 min in a digital thermostat water bath (HH-4, Instrument Manufacturing Co., Ltd., Rongxing, Changzhou, China). After incubation, 70 μL of EDTA was added to stop the enzymatic reactions. The ZnPP-formation ability after incubation was determined using the multifunctional enzyme marker, as described in Section 2.2.3. To quantify the ZnPP concentration, a standard curve equation for ZnPP (0.319 to 0.958 μM) was derived by diluting ZnPP (1.60 mM) in THF with a buffer solution containing tris-HCl (59 mM), 5% glycerol (*w*/*v*), 0.2% KCl (*w*/*v*), 48% ethanol (*v*/*v*), and 0.25% Triton X-100 (*w*/*v*). The standard curve equation (*R*^2^ = 0.9990) can be expressed as

(1) $$ZnPP=\frac{F-464.67}{53,891}\;$$

where *F* is the fluorescence intensity and ZnPP is the concentration of extracts (nm/mL). This linear relationship was used to calculate the bacterial intracellular and extracellular FECH activities. One activity unit was defined as a milliliter of solution that catalyzed 1 nmol ZnPP in 1 h.

### 2.4. Construction of Raw Minced Meat Model System

Raw minced meat was prepared from the fresh pork, with the surface trimmed using a sterilized knife and the meat minced using a sterilized meat grinder. The meat was mixed with 3.0% NaCl based on meat weight. Vacuum packing bags were sterilized with UV light overnight on a sterile workbench. Five groups (in triplicate) were formulated: a control group, a nitrite group with 0.1 g/kg sodium nitrite, and three bacteria-inoculated groups inoculated with high ZnPP-forming bacteria at levels of 10^7^ CFU/g meat. Samples were vacuum-packed in sterile bags and incubated at 25 °C for 0, 1, 3, and 5 d. A total of 120 meat samples were prepared, and the weight of each sample was 50 g. Each treatment had 24 samples. At each storage time for each treatment, 6 samples were used. Among these, 3 samples were used to analyze the physicochemical properties and color of raw minced meat, and another 3 samples were heated to analyze the color of cooked minced meat. The instrumental color and visible images of raw minced meat and cooked meat were examined, along with the pH, fluorescence, and absorption spectra of the raw minced meat. The whole experiment was repeated three times.

### 2.5. Instrumental Color and Visible Images

To compare the color of minced meat subjected to different treatments, the instrumental color and visible images of raw minced meat and cooked minced meat were analyzed. At each determination time, 6 samples were used: 3 samples were analyzed for the color of raw minced meat, and another 3 samples were analyzed for the color of cooked minced meat. For raw meat samples, the vacuum bags were opened and the interior color of the sample was determined. For the cooked minced meat, the vacuum bags were heated at 75 °C for 15 min in the digital thermostat water bath (HH-6, Changzhou Aohua Instrument Co., Ltd., Changzhou, China) and sample interior color was determined. The instrumental color was determined using a ZE-6000 colorimeter with 50 mm aperture, C illuminant, and 20 standard observer (Nippon Denshoku, Kogyo Co., Tokyo, Japan). The results are presented in terms of L*-, a*-, and b*- values, which represent the lightness, redness, and yellowness of samples, respectively. The instrument was calibrated with a white standard plate (L* = 95.26, a* = −0.89, b* = 1.18). Measurements for each treated sample were obtained three times, with the sample rotated 120° between measurements. In an enclosed space, with a lightless environment relying solely on fixed lighting, a digital camera steadily took a fixed-position image photo vertically down (HONOR 90, Zhengzhou, China).

### 2.6. pH Measurement

The pH of minced meat was measured according to the described method by Chen et al. [27]. Specifically, 10 g of minced meat from each group was mixed with 90.0 mL of distilled water. The mixture was vortexed, allowed to stand for 30 min, and then filtered. The pH of the filtrate was measured at 25 °C using a pH meter (FE20, Mettler-Toledo Instruments Co., Ltd., Shanghai, China). The pH meter was calibrated with manufacturer-provided standard solutions (pH 4 and 7) before use.

### 2.7. Fluorescence Spectroscopy of ZnPP in Minced Meat Extracts

The ZnPP in minced meat was extracted according to the method described by Wu et al. [28] with slight modifications. Briefly, 3 g of minced meat was mixed with 15 mL 75% acetone. After stirring, the tubes were placed at 4 °C under dark conditions for 30 min, then centrifuged at 10,619× *g* for 5 min at 4 °C. The supernatant was filtered using No. 2 filter paper (Toyo Roshi Kaisha Ltd., Tokyo, Japan). The fluorescent spectra of the filtrate were analyzed from 540 to 670 nm with an excitation at 420 nm using a fluorescence spectrophotometer (*RF6000, Shimadzu Production Co., Ltd., Tokyo, Japan) at a scanning speed of 600 nm/min. The fluorescence intensity at 590 nm (excitation at 420 nm) was used to assess the formation ability of ZnPP. 

### 2.8. UV–Vis Absorption Spectroscopy

The supernatant extracted with 75% acetone (as described in Section 2.7) was used for UV–Vis absorption spectroscopy analysis [29]. Absorption spectra were recorded at 400–600 nm with a double-beam spectrophotometer (UT1810, Purkinje General Instrument, Ltd., Beijing, China).

### 2.9. Statistical Analysis

The experimental results, presented as mean ± standard error (SE), were analyzed using the SPSS Statistics 22.0 software package (SPSS Inc., Chicago, IL, USA). The statistical significance was evaluated at the *p* < 0.05 level through the use of one-way and two-way analysis of variance, along with the application of Duncan’s multiple-range test. Each experiment was performed three times as separate trials, and every trial was replicated three times. A mixed model was used to study the ZnPP-forming ability of LAB, considering different strains and replicates as fixed and random terms, respectively. In analyzing the intracellular and extracellular enzymatic activity of the three ZnPP-forming LAB, the three strains and location of the FECH served as the fixed terms, while each replicate was treated as a random term. Variations in the physicochemical properties of samples subjected to different treatments and spectra of acetone extracts were derived using a mixed procedure where sample types (control group, nitrite group, *W. viridescens* JX11 group, *W. viridescens* MDJ8 group, and *L. Pentosus* Q group), fermentation time, and their interaction were considered fixed effects, and each replicate was considered a random effect. With the aid of Origin Pro 2021, the graphs were prepared (Origin Lab Co., Northampton, MA, USA).

## 3. Results and Discussion

### 3.1. Screening of ZnPP-Forming LAB 

**Figure 1:**
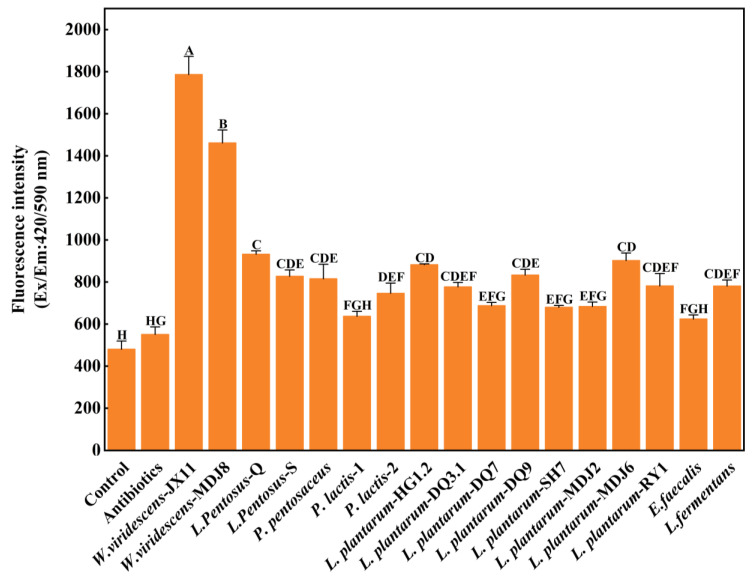
Fluorescence intensity of the aseptic ZnPP-formation model system at 25 °C for 3 d, including the control group (no LAB inoculation), antibiotics group (antibiotic addition), and LAB inoculated groups (final conc. × 10^7^ CFU/mL). The 17 strains of bacteria were obtained from various food sources. Uppercase letters (A–H) indicate significant differences between different LAB (*p* < 0.05).

Fluorescence intensity is an indicator of the formation ability of ZnPP, with a higher intensity corresponding to stronger ZnPP-forming ability [26]. Figure 1 shows the fluorescence intensity of ZnPP extracts from 17 LAB in an aseptic model experimental system. There was no notable difference in fluorescence intensity detected between the control and antibiotic groups (*p* > 0.05), suggesting that ZnPP formation was significantly inhibited in the control group, thus confirming the aseptic conditions of the control group during model preparation. After incubation, the fluorescence intensity pertaining to the inoculated groups was significantly higher than that of the control except for *Enterococcus faecalis* and *Pediococcus lactis*-1, and significantly higher than that of the antibiotic group except for *L. plantarum* DQ7, *L. plantarum* SH7, *L. plantarum* MDJ2, *E. faecalis*, and *P. lactis-1*. The fluorescence intensity of *W. viridescens* JX11 and *W. viridescens* MDJ8 groups significantly exceeded that of other inoculated groups (*p* < 0.05). In particular, the fluorescence intensity of *W. viridescens* JX11 was approximately two times that of the other inoculated groups, except for *W. viridescens* MDJ8. A significantly lower fluorescence intensity was observed for *L. Pentosus* Q compared to the *W. viridescens* JX11 and *W. viridescens* MDJ8 groups (*p* < 0.05), whereas it was notably higher than the fluorescence intensity of the remaining inoculation groups. In addition, some LAB strains from different regions showed varying ZnPP-forming abilities, such as *W. viridescens* JX11, and *W. viridescens* MDJ8. Based on the ZnPP-forming abilities, *W. viridescens* JX11, *W. viridescens* MDJ8 and *L. Pentosus* Q were selected for further investigation.

### 3.2. FECH Activity Analysis

**Figure 2:**
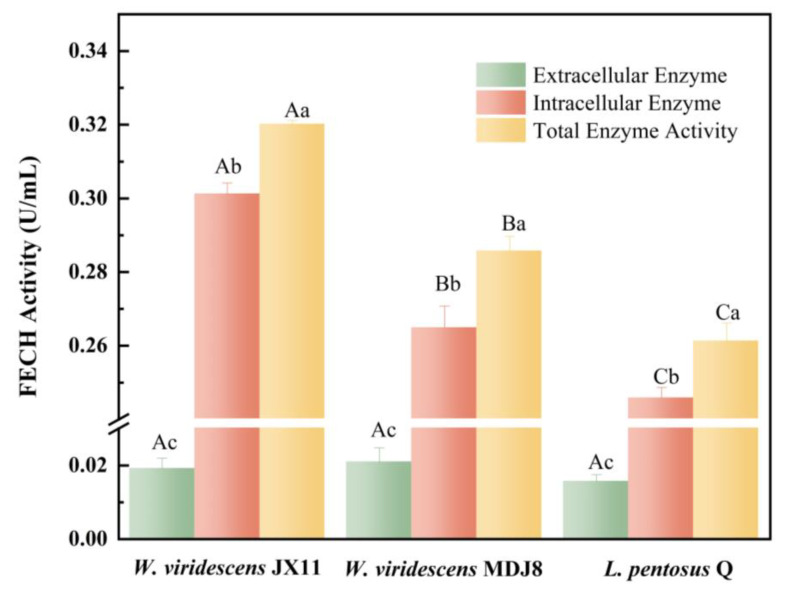
FECH activity of *W. viridescens* JX11, *W. viridescens* MDJ8, and *L. pentosus* Q. Bacteria were incubated for 16 h at 37 °C. Different uppercase letters (A–C) indicate significant differences between the different LAB inoculation groups, and lowercase letters (a–c) indicate significant differences within the same LAB inoculation group (*p* < 0.05).

ZnPP is believed to be produced by meat-inherent and microbial FECH [19]. FECH, serving as a terminal enzyme in the heme biosynthesis pathway, facilitates the insertion of Fe^2+^ into protoporphyrin IX (PPIX), resulting in the formation of heme. Additionally, it can catalyze the insertion of Zn^2+^ into PPIX, leading to the production of ZnPP under specific conditions [14,30,31]. The FECH activity of the bacteria was inspected to assess their ability to produce ZnPP. Figure 2 illustrates the intracellular, extracellular, and total FECH activity of *W. viridescens* JX11, *W. viridescens* MDJ8, and *L. pentosus* Q. The extracellular enzyme activity of the three bacteria was extremely low, while the intracellular enzyme activity of all strains was significantly higher than the extracellular enzyme activity (*p* < 0.05), indicating that FECH is predominantly an intracellular enzyme. The intracellular FECH activity of *W. viridescens* JX11 (0.301 U/mL) was significantly higher than that of *W. viridescens* MDJ8 (0.265 U/mL) and *L. pentosus* Q (0.240 U/mL) (*p* < 0.05). 

### 3.3. Color Analysis and Visible Images

**Figure 3:**
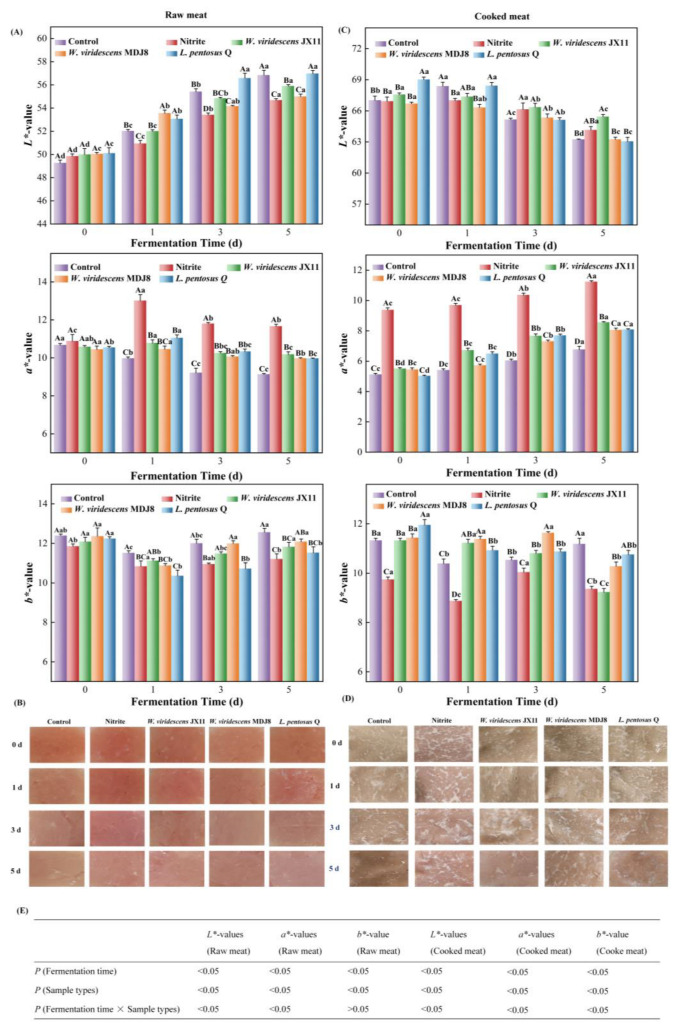
Influence of three strains of high ZnPP-forming LAB on instrumental color and visual appearance of minced pork during fermentation at 25 °C. (**A**–**D**) Instrumental color and visible images of raw minced meat and cooked minced meat heated at 75 °C for 15 min. (**E**) The interaction of fermentation time and sample types. Uppercase letters (A–C) indicate significant differences between the different groups at the same fermentation time, and lowercase letters (a–d) indicate significant differences between the different fermentation times within a treatment group (*p* < 0.05).

The effect of ZnPP-forming bacteria on the instrumental color and visible images of minced meat is presented in Figure 3. Figure 3E reveals significant effects of the interaction between fermentation time and sample types on the instrumental color attributes of meat, excluding the b*-value of raw meat (*p* < 0.05). L*-values of raw minced meat increased with fermentation time in all groups, likely due to the breakdown of carbohydrates, accumulation of organic acids as well as denaturation of protein [21]. The L*-values of the *W. viridescens* JX11 and *L. pentosus* Q groups were significantly higher than that of the nitrite group (*p* < 0.05), with no significant difference between the *W. viridescens* MDJ8 group and the nitrite group after 5-d fermentation (*p* > 0.05) (Figure 3A). The a*-value of the control group decreased over time, whereas those of the nitrite group and all bacteria-inoculated groups increased and then decreased. The a*-values of all bacteria-inoculated groups were lower than those of the nitrite group, but significantly higher than that in the control group after 3- and 5-d fermentation (*p* < 0.05). The red color of the nitrite group was primarily due to the generation of nitrosomyoglobin (NOMb), whereas the red color of the bacteria-inoculated group could be attributed to the formation of ZnPP [11,32]. Except for the *W. viridescens* MDJ8-inoculated group, the b*-value of the bacteria-inoculated groups and nitrite group was significantly lower than that of the control group after 5-d fermentation (*p* < 0.05). The outcomes were in line with the visual images (Figure 3B). No obvious difference was observed among the visual images of all groups at the beginning of fermentation. By day 5, the *W. viridescens* JX11-inoculated and *L. pentosus* Q-inoculated groups had a cherry-red color similar to the nitrite group and were much more vibrant than the control group. The color of the control group was visually duller than those of other groups at the end of fermentation, suggesting that high ZnPP-forming bacteria could potentially replace or partially replace nitrite. 

To assess the heat stability of the red pigment, the color of cooked minced meat (heated at 75 °C for 15 min) was analyzed (Figure 3C). The L*-values of cooked minced meat decreased over fermentation time in all groups. The a*-value of the control group was the lowest during the fermentation period, especially at the end, where the b*-value was the highest at 5-day fermentation. Therefore, the color of the control group was noticeably duller in comparison to the other groups (Figure 3D). The a*-values of cooked meat in the bacteria-inoculated groups were markedly higher than that of the control group and lower than that of the nitrite group (*p* < 0.05). The a*-value of *W. viridescens* JX11 was the highest, except for the nitrite group after 5-d fermentation, followed by the *W. viridescens* MDJ8-inoculated and *L. pentosus* Q-inoculated groups, consistent with the visual images (Figure 3D). The color of bacteria-inoculated groups remained stable to a certain extent, indicating the thermal stability of the red pigment. Although the a*-value of the bacteria-inoculated groups is higher than that of the control group without nitrite, the fact that it is lower than that of the nitrite-treated groups indicates these specific LAB strains may be more suitable for dry fermented products than for cooked meat products. 

### 3.4. pH Changes

**Figure 4:**
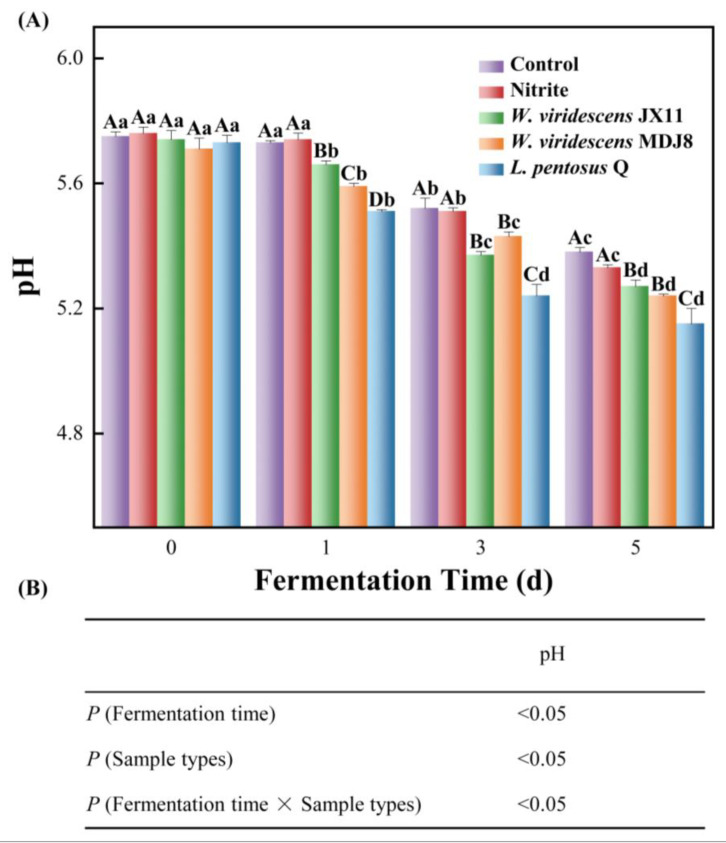
Influence of three strains of high ZnPP-forming LAB on the pH of minced pork during fermentation at 25 °C (**A**). (**B**) The interaction of fermentation time and sample types. Uppercase letters (A–C) indicate significant differences between different groups at the same fermentation time, and lowercase letters (a–d) indicate significant differences between different fermentation times within a treatment group (*p* < 0.05).

The pH of fermented meat products varies according to the type of starter and the time of fermentation, so pH is an important factor affecting the formation of ZnPP [33]. The pH of minced meat in all groups showed a decreasing trend with increased fermentation time (Figure 4), consistent with previously reported results [34,35]. Figure 4B illustrated that the pH of minced meat was significantly affected by fermentation time, sample types, and their interaction (*p* < 0.05). Initially, all minced meat samples had an initial pH of approximately 5.7. After a five-day fermentation process, the pH of the control, nitrite, *W. viridescens* JX11, *W. viridescens* MDJ8 and *L. pentosus* Q samples dropped significantly to 5.38, 5.33, 5.27, 5.24, and 5.15, respectively (*p* < 0.05). The inoculated groups exhibited a more rapid pH decline compared to the control and nitrite groups, likely due to the metabolism of carbohydrates by LAB, which grew and multiplied, yielding organic acids such as lactic and acetic acids, thereby lowering the pH [36]. The *L. pentosus* Q group exhibited a notably lower pH compared to the other groups (*p* < 0.05), indicating stronger acid production ability. This property is beneficial for meat products, as a lower pH can efficiently inhibit pathogenic and spoilage-inducing microorganisms [37]. At the end of fermentation, the fluorescence intensity of ZnPP in *L. pentosus* Q group was significantly lower than that in the other two bacteria-inoculated groups, indicating that the formation of ZnPP may be more obvious in a proper pH environment for ZnPP-formation bacteria, considering the effect of pH on FECH [38]. As proved in Figure 2, FECH activity of *L. Pentosus* Q was significantly lower than that of *W. viridescens* JX11 and *W. viridescens* MDJ8.

### 3.5. Fluorescence Spectrum Analysis

**Figure 5:**
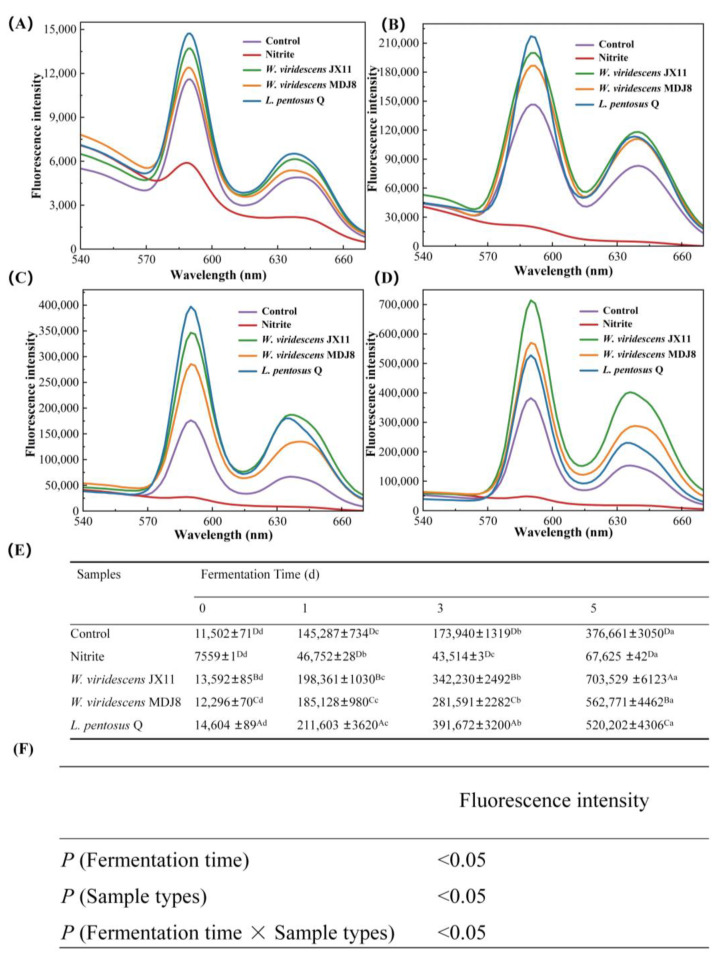
Influence of three strains of high ZnPP-forming LAB on the fluorescence spectra of ZnPP extracts from salted minced pork during fermentation at 25 °C. (**A**–**D**) Fermentation for 0, 1, 3, and 5 d, respectively. (**E**) The results of fluorescence intensity analysis (*E_x_* = 420 nm, *E_m_* = 590 nm). (**F**) The interaction of fermentation time and sample types. Uppercase letters (A–D) indicate significant differences between the different treatment groups at the same fermentation time, and lowercase letters (a–d) indicate significant differences between different fermentation times within a treatment group (*p* < 0.05).

ZnPP stands out as a red substance characterized by a prominent fluorescence peak occurring at an excitation wavelength of 420 nm and an emission wavelength of 590 nm [24,39], easily distinguishable from NOMb, which lacks fluorescence. Figure 5F reveals that the fluorescence intensity of ZnPP extracts was significantly affected by the fermentation time, sample types, and their interaction (*p* < 0.05). According to the fluorescent spectra shown in Figure 5A–D, all groups exhibited very low fluorescence peaks of ZnPP at day 0. During the fermentation process, the fluorescence peak of all groups significantly increased, except for the nitrite group, consistent with previous reports that indicate that nitrite may suppress ZnPP formation to a certain extent [40]. 

The ZnPP content in the minced meat was assessed by the intensity of the peaks. According to the table in Figure 5E, the fluorescence intensity of the bacteria-inoculated groups was significantly higher than that of the control group (*p* < 0.05), consistent with the a*-values (Figure 3). The fluorescence intensities observed in the three inoculated groups were significantly higher compared to those of the control and nitrite groups, with the control group displaying the lowest fluorescence intensity, throughout the fermentation (*p* < 0.05). The *L. pentosus* Q group showed the highest fluorescence intensity until day 3, but by day 5, it exhibited the lowest intensity among the inoculated LAB strains (*p* < 0.05); and the *W. viridescens* JX11 groups exhibited the highest fluorescence intensity, followed by *W. viridescens* MDJ8 and *L. pentosus* Q at day 5 (*p* < 0.05). Those results may be due to the adaptability of different strains to culture conditions. The effect of pH on FeCH activity as described above affects the formation of ZnPP [38]. Wakamatsu, Kawazoe, Ohya, Hayakawa, and Kumura [41] found the ideal pH for ZnPP formation in pork was pH 5.5. In this study (Figure 4), the *W. viridescens* JX11 group exhibited the highest ZnPP levels at pH 5.27 for 5 days of fermentation. Additionally, the strain *W. viridescens* JX11, which had the highest FeCH activity, showed a low fluorescence intensity on days 0, 1, and 3, and the highest fluorescence intensity on day 5, which may be due to that on day 5, *W. viridescens* JX11 group were still at a proper pH environment for FeCH activity These results indicate that the inoculated LAB significantly contributed to ZnPP formation, enhancing the brightness and redness of the meat color. In addition, the enhanced fluorescence intensity observed in the control group could stem from the activity of endogenous meat enzymes, notably FECH [19,30,42]. The lower fluorescence intensity in the nitrite group suggested that the addition of nitrite led to a decrease in the formation of ZnPP, while bacterial inoculation greatly promoted the formation of ZnPP, thereby improving the color of minced meat.

### 3.6. Absorption Spectra Analysis

**Figure 6:**
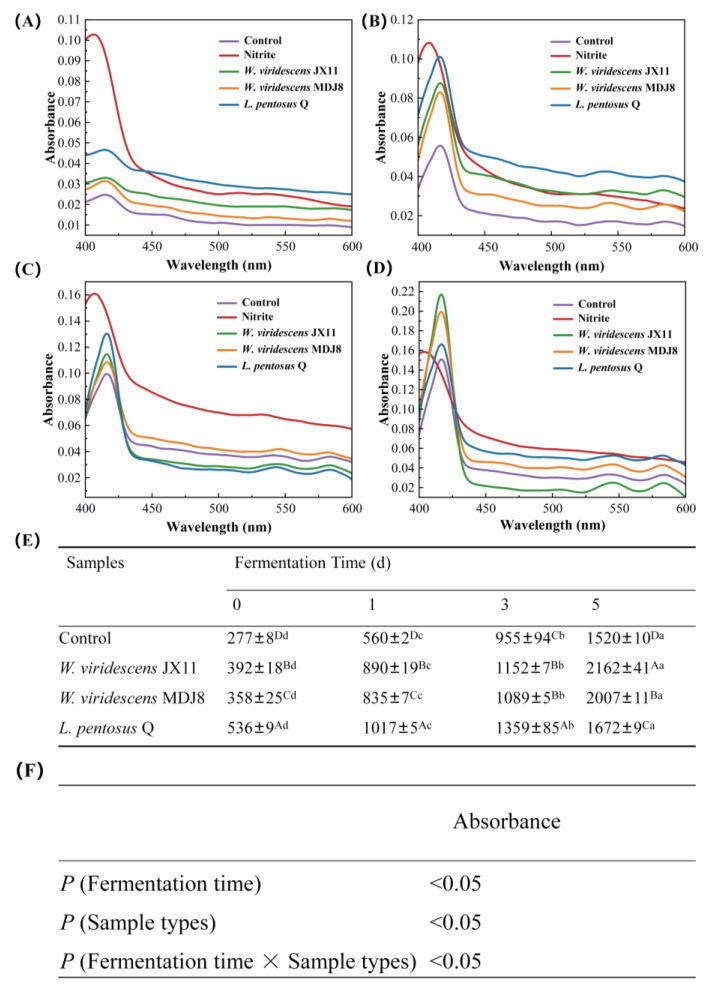
Influence of three strains of high ZnPP-forming LAB on UV–Vis absorption spectra of ZnPP extracts from minced pork during fermentation at 25 °C. (**A**–**D**) Fermentation for 0, 1, 3, and 5 d, respectively. (**E**) The absorption intensity results at 417 nm (Abs × 10^−4^). (**F**) The interaction of fermentation time and sample types. Uppercase letters (A–D) indicate significant differences between the different groups at the same fermentation time, and lowercase letters (a–d) indicate significant differences between the different fermentation times within a treatment group (*p* < 0.05).

In addition to fluorescence measurement, spectrophotometric data can also serve as a valuable tool for comprehending the chemical properties of pigments and reflecting the forms of myoglobin derivatives in the extract [29]. The absorption spectra of ZnPP extracts from minced pork during fermentation are presented in Figure 6, exhibiting a trend similar to the fluorescence spectra (Figure 5). The UV absorption peak of the nitrite group, distinguished by its special red pigment NOMb, differed from the other groups, with no special peak of ZnPP observed at 417 nm. The characteristic peak of NOMb in the UV spectrum has been reported to be approximately 395 nm [43]. However, in Figure 6A–C, the Soret band of the nitrite group is not at 395 nm but rather shifted above 400 nm. A plausible explanation could be attributed to the presence of a complex mixture of Mb derivatives in meat samples and the effects of pH on the NOMb state, which lead to the rightward shift of Soret band of the nitrite group [35,44]. As shown in Figure 6F, the interaction between the fermentation time and sample types exhibited significant effects on the absorbance of ZnPP extracts (*p* < 0.05). The UV–Vis absorbance data at 417 nm in Figure 6E indicated that inoculated groups exhibited higher absorbance compared to the control group (*p* < 0.05), with the *W. viridescens* JX11 group at day 5 exhibiting the highest absorbance, consistent with the fluorescence intensity results (Figure 5E). As the fermentation time increased, the UV–Vis absorbance of all groups increased (*p* < 0.05).

## 4. Conclusions

In this study, the high ZnPP-forming LAB were screened and their FECH activity and color-enhancing ability in a minced-meat model were evaluated. *W. viridescens* JX11 exhibited significantly higher FECH activity than the other two strains, and the intracellular FECH activity of all bacteria was higher than the extracellular enzyme activity. When these three bacteria were inoculated into minced meat, although the bright red color observed in the bacteria-inoculated groups was not as good as that of the nitrite group, it was significantly better than that of the control group, especially for *W. viridescen* JX11, which showed its potential as the nitrite alternative. The LAB strains screened in cooked meat did not show a*-values comparable to that of the nitrite group. Therefore, these specific LAB strains may be more suitable for dry fermented products than for cooked meat products. The fluorescence intensity and UV–Vis absorbance analysis revealed that the incorporation of LAB contributed to the formation of ZnPP in the fermented minced meat. Overall, the use of LAB, especially *W. viridescens* JX11, has the potential to enhance the color of meat products. These findings provide a new avenue for exploring nitrite substitutes.

## Figures and Tables

**Table 1:** Type and sources of lactic acid bacteria.

Bacterial Name	Type	Source
*Weissella viridescens*	*W. viridescens* JX11	Dry sausage
	*W. viridescens* MDJ8	
*Lactobacillus pentosus*	*L. pentosus* Q	Shanghai Bioresources Collection Center
	*L. pentosus* S	
*Pediococcus pentosaceus*	*P. pentosaceus*	Dry sausage
*Pediococcus lactis*	*P. lactis-1*	
	*P. lactis-2*	
*Lactobacillus plantarum*	*L. plantarum* HG1.2	Sauerkraut
	*L. plantarum* DQ3.1	
	*L. plantarum* DQ7	Dry sausage
	*L. plantarum* DQ9	
	*L. plantarum* SH7	
	*L. plantarum* MDJ2	
	*L. plantarum* MDJ6	
	*L. plantarum* RY1	
*Enterococcus faecalis*	*E. faecalis*	
*Lactobacillus fermentans*	*L. fermentans*	

## Data Availability

The original contributions presented in the study are included in the article, further inquiries can be directed to the corresponding author.
